# Short-Term Relationship Between Air Pollution and Mortality from Respiratory and Cardiovascular Diseases in China, 2008–2020

**DOI:** 10.3390/toxics13030156

**Published:** 2025-02-24

**Authors:** Yunning Liu, Xuyang Shan, Yitong Sun, Xinpeng Guan, Lijun Wang, Xinghou He, Jiangmei Liu, Jinling You, Rongshan Wu, Jianbin Wu, Bin Zhang, Jinlei Qi, Peng Yin, Mengyao Li, Xinghua He, Zifa Wang, Hongbing Xu, Jing Wu, Wei Huang

**Affiliations:** 1 National Center for Chronic and Noncommunicable Disease Control and Prevention, Chinese Center for Disease Control and Prevention, Beijing 100050, China; liuyunning@ncncd.chinacdc.cn (Y.L.); sunyt0824@126.com (Y.S.); wanglijun@ncncd.chinacdc.cn (L.W.); liujiangmei@ncncd.chinacdc.cn (J.L.); youjinling@ncncd.chinacdc.cn (J.Y.); qijinlei@ncncd.chinacdc.cn (J.Q.); yinpeng@ncncd.chinacdc.cn (P.Y.); 2 Department of Occupational and Environmental Health, Peking University School of Public Health, Peking University Institute of Environmental Medicine, Beijing 100191, China; shanxuyang@pku.edu.cn (X.S.); a3ziyuezhishou@163.com (X.G.); 2111110178@bjmu.edu.cn (X.H.); 2211110213@bjmu.edu.cn (B.Z.); 1710306109@pku.edu.cn (M.L.); xuhongbing@bjmu.edu.cn (H.X.); 3 State Key Laboratory of Vascular Homeostasis and Remodeling, Peking University, Beijing 100871, China; 4 State Key Laboratory of Environmental Criteria and Risk Assessment, State Environmental Protection Key Laboratory of Ecological Effect and Risk Assessment of Chemicals, Chinese Research Academy of Environmental Sciences, Beijing 100012, China; rongshanwu@bjmu.edu.cn; 5 School of Geography, Nanjing Normal University, Nanjing 210023, China; wujianbin83@126.com (J.W.); 211302028@njnu.edu.cn (X.H.); 6 State Key Laboratory of Atmospheric Boundary Layer Physics and Atmospheric Chemistry, Institute of Atmospheric Physics, Chinese Academy of Sciences, Beijing 100029, China; zifawang@mail.iap.ac.cn

**Keywords:** short-term, air pollution, respiratory diseases, CVDs, mortality, case crossover study

## Abstract

Most existing epidemiological studies on the impact of air pollution on noncommunicable diseases have focused on urban areas, rather than nationwide studies that include rural areas. This study utilized a time-stratified case-crossover study that included deaths registered in the National Mortality Surveillance System from 2008 to 2020. Atmospheric particulate matter (PM_10_ and PM_2.5_), nitrogen dioxide (NO_2_), sulfur dioxide (SO_2_), and carbon monoxide (CO) were evaluated via the National Nested Air Quality Prediction Modeling System. Conditional logistic regression was used to assess the associations between short-term air pollution exposure and the risk of respiratory disease and cardiovascular disease (CVD) mortality. There were increases in the risk of respiratory diseases (0.12%, 0.10%, 0.10%, 0.05%, and 0.40%) and CVDs (0.08%, 0.07%, 0.03%, 0.02%, and 0.22%) for each 10 μg/m^3^ increase in the concentrations of PM_10_, PM_2.5_, NO_2_, and SO_2_, respectively, and for each 1 mg/m^3^ increase in the concentration of CO, which may be associated with the participants’ characteristics. The results of these national analyses indicate that ambient air pollutants are significantly associated with increased risks of respiratory disease and CVD death in both urban and rural areas, which is critical for air pollution control, especially in low- and middle-income areas.

## 1. Introduction

Air pollutants are complex mixtures of solids and gases, which are one of the most important public health problems and affect the health of almost the entire global population [1]. Air pollution is recognized as the leading environmental risk factor for mortality and morbidity worldwide. The Global Burden of Disease 2021 study revealed that air pollution is a major contributor to the global burden of disease and is one of the top 10 risk factors for the global burden of disease in children and the elderly [2]. Many studies have shown that environmental pollution increases the risk of death from respiratory diseases and cardiovascular diseases (CVDs) [1,3]. There is growing concern worldwide about the increased risk of death from chronic noncommunicable diseases caused by air pollution.

To protect human health, the World Health Organization (WHO) released a new version of its air quality guidelines (AQGs) in 2021, limiting pollutants to more stringent limits [4]. Numerous regulations and measures have been enacted to limit the emission of air pollutants. Moreover, numerous scientific studies have demonstrated the relationship between short-term exposure to air pollutants and increased deaths from chronic noncommunicable diseases. However, the evidence in support of the current WHO pollutant concentration limits is insufficient in developing countries. Probably because of the difficulty of obtaining national monitoring data, researchers usually conduct studies only on representative large, economically developed cities, ignoring the wide range of smaller cities and rural areas. Most prior studies reported findings on a few selected pollutants (e.g., PM_2.5_) in association with cause-specific mortality, raising concerns about potential publication bias. Air pollution, a serious environmental problem, often has adverse effects on the respiratory and cardiovascular systems. Therefore, there is an urgent need for nationwide studies on the relationships between air pollutants and deaths from respiratory diseases and CVDs in developing countries.

Currently, China’s National Ambient Air Quality Standards (NAAQSs) are still largely based on WHO-recommended AQGs. Air quality in China has improved significantly over the past decade as a result of effective management and scientific monitoring, and it is unknown whether an update of the NAAQSs is needed due to changes in air quality and population susceptibility. Therefore, using a time-stratified case-crossover study approach, this study analyzed the associations between short-term exposure to five major air pollutants (particulate matter with diameters less than 2.5 and 10 μm, PM_2.5_ and PM_10_; nitrogen dioxide, NO_2_; sulfur dioxide, SO_2_; carbon monoxide, CO) and deaths from respiratory diseases and CVDs in the population on a national scale.

## 2. Materials and Methods

### 2.1. Data Source and Quality Control

This study used mortality data from the National Mortality Surveillance System (NMSS) of the Chinese Center for Disease Control and Prevention (CDC), which is nationally representative. Owing to air pollutant data limitations, two provinces, Tibet and Xinjiang, were not included in the present analysis. The NMSS conducts regular under-reporting surveys every year. The provincial CDC continuously collects death information from various sources, such as public security and civil affairs, from various departments and conducts information checking. The national CDC then adds to the reported under-reported cases, removes duplicate data, and logically verifies the coding of potential causes of death. These operations ensure data completeness and accuracy. The deaths were classified according to the International Classification of Diseases 10th revision (ICD-10; as shown in Supplementary Table S1).

### 2.2. Environmental Assessment

Five air pollutants are involved in this study, including PM_2.5_, PM_10_, NO_2_, SO_2_, and CO. The daily mean concentrations of the pollutants at 15 km (km) spatial resolution are generated via the nested grid air quality prediction model system (NAQPMS) simulation, and township-level exposures were estimated for each participant based on their residential address. The NAQPMS was developed independently by the Chinese Academy of Sciences (CAS) as a system for the comprehensive assessment of 3-D regional Eulerian numerical air quality models at regional and urban scales. The NAQPMS consists of four subsystems, namely, the basic data system, the weather forecasting system, the air pollution forecasting system, and the forecast result analysis system. The system is an important tool for studying the interactions between pollutant emissions, meteorological conditions, chemical transformations, and wet and dry removal. It consists of a meteorological treatment module and a chemical transport module and performs simulation and evaluation work by combining the emission, transport, and evolution characteristics of complex atmospheric pollution in Chinese urban agglomerations [5]. NAQPMS utilizes real monitoring information from air quality testing stations as the source of data underlying the subsystem. In addition, the system utilizes actual data from ground monitoring stations to synthesize and simulate air quality data, thus improving the accuracy of the model simulation process [6]. The air pollutant exposure levels estimated using the model may be biased from the real data, but comparative studies have shown that the errors are still within manageable limits [7]. Researchers pointed out that the nationwide air pollution simulation data based on the NAQPMS model had a strong correlation with the ground monitoring data, and the correlation coefficient between the two was greater than 0.9 during 2015–2018 [8]. NAQPMS can provide scientific pollution emission control countermeasures for environmental decision-making.

Meteorological data include daily ambient temperature and relative humidity data, which were collected from the monitoring data of the National Meteorological Service (http://data.cma.cn/ (accessed on 20 December 2024)). The monitoring system includes 2171 monitoring stations throughout the country. We also applied meteorological data measured at the nearest monitoring point for each participant by matching latitude and longitude to 41,636 townships, towns, and streets across the country.

### 2.3. Statistical Analysis

This study utilized a time-stratified case-crossover design. We used each participant himself/herself as his/her control to estimate the relationship between short-term exposure to air pollution and mortality from respiratory and CVDs. The day on which a case occurs is defined as a case day, and other days in the same year, month, and day of the week as the case day are defined as control days. In this way, the case days and control days are in the same time stratum. A case day can be randomly matched with multiple control days. The advantage of this design is that it facilitates controlling for the impact of long-term trends, seasonality, and day-of-the-week effects on this study. In addition, this design approach allows for the control of the potential effects of characteristics at the individual level (e.g., age, gender, pre-existing disease, health-related behaviors, and socioeconomic status) and risk factors that do not change much in the short term among study participants (e.g., body mass index and indoor air pollution) [9].

For the analysis of the associations of short-term exposure to air pollution with population-specific causes of death, we used a conditional logistic regression model to analyze the associations between deaths from respiratory diseases and CVDs and short-term exposure to air pollutants in each province. All regression models were adjusted for potential confounding impacts of weather conditions via natural spline functions with 6 and 3 degrees of freedom for 3-day moving averages of temperature and relative humidity, respectively [10]. Most previous studies have shown that there is a delayed exposure period for the effects of short-term exposure to air pollutants on deaths from respiratory diseases and CVDs in the population [11,12]. Therefore, we focused on the delayed effects of the first 2 days in this study. Specifically, the lag patterns included single-day lag 0 (the same day) to lag 2 (two days before death), as well as cumulative lag effects lag 01 (the mean between the same day of death and the prior day 1) and lag 02 (the mean between the same day of death and the prior day 2).

Several analyses were subsequently performed to determine the susceptibility of different individuals and to verify the robustness of the results, with effect values stratified according to sex, age, season, and residential area of the study population. There were four age groups (<65 years, 65–74 years, 75–84 years, and ≥85 years) [13]. The seasons were categorized into two groups: the cold season (November–March) and the warm season (April–September). The residential areas were categorized into two groups: urban and rural. We tested the significance of the differences in effect estimates between strata by calculating 95% confidence intervals with the following formula: $\left(b_{1}-b_{2}\right)\pm 1.96\sqrt{{\left(SE_{1}\right)}^{2}+{\left(SE_{2}\right)}^{2}}$ (*b*_1_ and *b*_2_ are effect estimates for each stratum, and *SE*_1_ and *SE*_2_ are standard errors) [14].

In addition, to evaluate the stability of the health effects of short-term exposure to individual pollutants, this study conducted sensitivity analyses by constructing a two-pollutant model. The confounding effects of copollutants were assessed by adjusting for the second pollutant in the single-pollutant model.

We chose to report the percentage change in mortality risk due to a one-unit increase in the concentration of each pollutant (scaled to 10 μg/m^3^ for PM_2.5_, PM_10_, NO_2_, and SO_2_ and 1 mg/m^3^ for CO). This allows for subsequent discussion of the results and comparison with those of previous studies. The expression for the transformation equation is as follows: $\left((e^{\left(b\;\times \;a\;unit\right)}-1\right)\times \;100\%)$ with 95% confidence intervals (CIs, lower 95%CI: $\left(\left(e^{\left(\left(b-1.96\;\times \;SE\right)\times \;a\;unit\right)}-1\right)\times 100\%\right)$, upper 95% CI: $\left((e^{\left(\left(b+1.96\times SE\right)\times \;a\;unit\right)}-1)\times \;100\%)\right)$. In the above equation, *b* is the regression coefficient (log OR), and *SE* is the standard error of the logit model. All analyses were performed via software version R4.2.2, and statistical tests were performed as two-sided probability values with a test level of α = 0.05.

## 3. Results

### 3.1. General Characteristics of the Study Population

A total of 7,171,833 respiratory diseases cases and 30,339,986 cardiovascular disease cases were included in this study. Table 1 shows the sex, age, area of residence, and cause of death of the study participants.

### 3.2. Air Pollution Exposure Levels

Table 2 presents the daily distributions of air pollution exposure levels on the case and control days for two disease classifications. Since the case days varied depending on different causes of death, the matched control days also differed accordingly. Therefore, the pollutant concentrations are presented in two groups (respiratory diseases and CVDs). The average pollutant concentration on the case days was slightly greater than that on the control days.

### 3.3. Percentage Increases in the Risk of Death from Respiratory Diseases and CVDs

**Figure 1:**
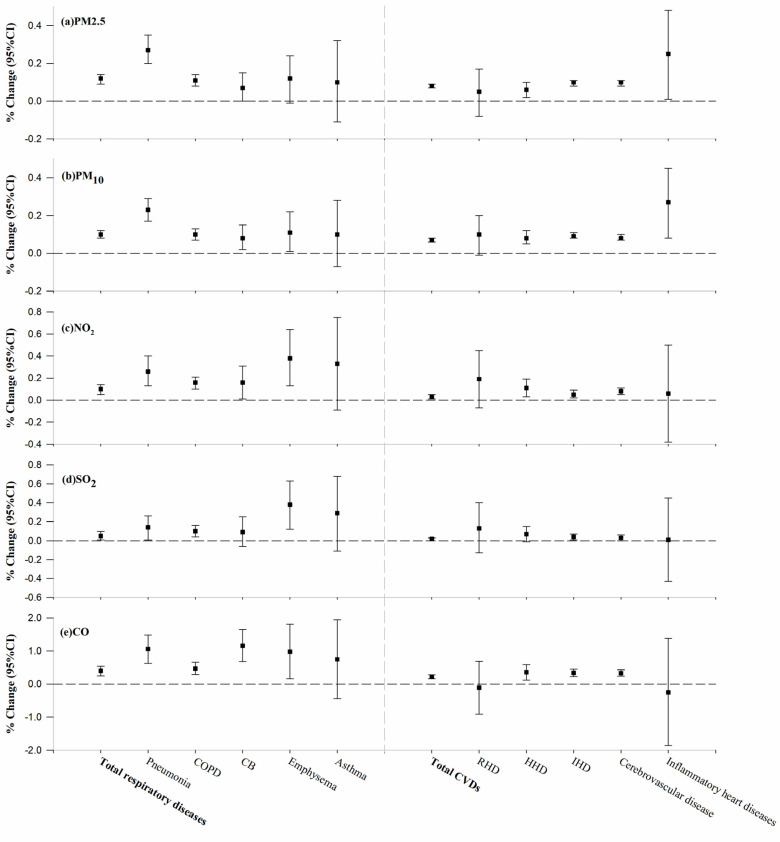
Percentage increases in the risk of death from respiratory diseases and CVDs on lag 0 due to short-term exposure to air pollutants.

Figure 1 summarizes the percentage increases in the risk of death from respiratory diseases and CVDs on lag 0 due to short-term exposure to air pollutants. We found that increased concentrations of all five common air pollutants (PM_2.5_, PM_10_, NO_2_, SO_2_, and CO) were associated with an increased risk of death from each of these diseases. On lag 0, each unit increase in pollutants (10 μg/m^3^ for PM_2.5_, PM_10_, NO_2_, and SO_2_, and 1 mg/m^3^ for CO) increased the risk of death from respiratory diseases by 0.12% (95% CI: 0.09, 0.14), 0.10% (95% CI: 0.08, 0.12), 0.10% (95% CI: 0.05, 0.14), 0.05% (95% CI: 0.01, 0.10), and 0.40% (95% CI: 0.25, 0.54) and increased the risk of death from CVDs by 0.08% (95% CI: 0.07, 0.09), 0.07% (95% CI: 0.06, 0.08), 0.03% (95% CI: 0.01, 0.05), 0.02% (95% CI: 0.00, 0.03), and 0.22% (95% CI: 0.16, 0.28). Short-term exposure to particulate matter pollution (PM_2.5_ and PM_10_) is associated with a relatively greater risk of death from pneumonia and inflammatory heart disease. The other three pollutants, however, had little effect on the risk of death due to inflammatory heart disease. The increased risk of death from NO_2_ and SO_2_ pollution was similar and was most pronounced for emphysema and asthma. Instead, the risk of death from chronic bronchitis increased the most with short-term exposure to CO pollution.

### 3.4. Stratified Analysis

**Figure 2:**
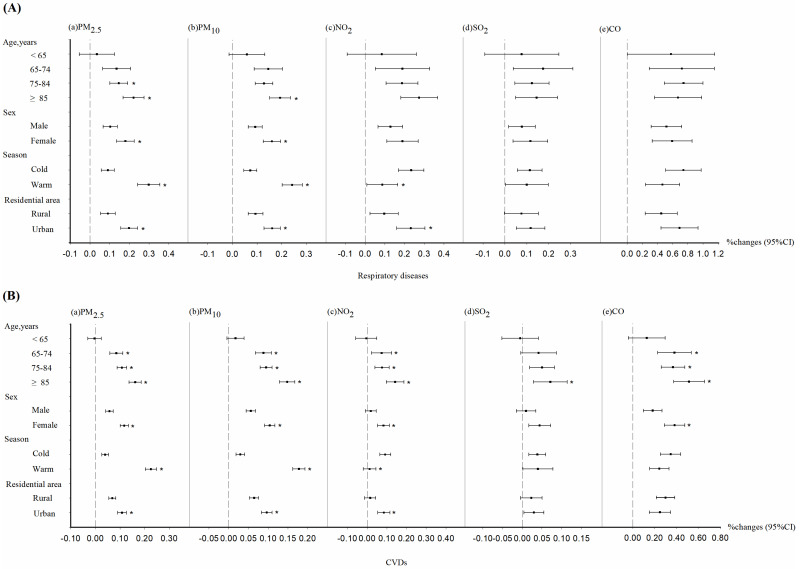
Percentage changes in mortality risks from respiratory (**A**) and CVDs (**B**) associated with increases in ambient pollutant concentrations stratified by the characteristics of the study participants. * *p* < 0.05.

Since we observed the most significant results at lag 0, we performed a further stratified analysis. Figure 2 shows the relationship between air pollution and mortality from respiratory diseases and CVDs, stratified by participant characteristics. Our observations indicated that the risk of death from CVDs due to air pollution increased with age for all five pollutants studied, while only PM_2.5_ and PM_10_ exhibited a similar trend in mortality from respiratory diseases. In addition, there was a significant sex difference in the risk of death from the two types of diseases caused by PM_2.5_ and PM_10_, with women having a higher risk of death, whereas NO_2_ and CO showed sex differences only in CVD-related deaths. PM_2.5_ and PM_10_ pose greater mortality risk increases in the warm season than in the cold season, whereas NO_2_ has the opposite effect. In terms of area of residence, participants in urban areas were more affected by PM_2.5_, PM_10_, and NO_2_ pollution than were those in rural areas for both respiratory diseases and CVDs. The effects of the remaining pollutants were not significant in terms of urban–rural differences.

### 3.5. Sensitivity Analysis

In this study, sensitivity analysis was performed by constructing a two-pollutant model. The results of the two-pollutant model shown in Table 3 do not significantly change the conclusions reached from the main model. This is due to the fact that when the correlation between two pollutants is extremely strong, it may lead to instability in the effects analysis. In this study, only two-pollutant models with correlation coefficients less than 0.7 in the Spearman rank correlation analysis are shown in the results [15]. In particular, we found that the effect estimates for PM_2.5_ and PM_10_ remained significant after adjusting for a second pollutant for both categories of disease. The effect estimates for CO exposure also remained significant after adjusting for SO_2_. When adjusting for SO_2_ pollution, the effect estimates for NO_2_ were only modestly changed in respiratory diseases. After adjusting for PM_2.5_ and PM_10_, the effects of the remaining pollutants (especially SO_2_ and NO_2_) were not significant or reduced. This might suggest that particulate matter (PM) is a relatively more important air pollution problem in China.

## 4. Discussion

Using monitoring data from 2008 to 2020, this study adopted a time-stratified case-crossover approach to analyze the associations between short-term exposure to five major air pollutants and deaths from respiratory diseases and CVDs among the population at the national level. We found that as the concentration of air pollutants increased, the risk of death from all types of diseases increased. The percentage increase in risk may differ depending on the characteristics of the participants, such as age, sex, season, and residential area. The results of the two-pollutant model showed that the effects of the remaining pollutants were not significant or reduced after adjusting for PM. This suggests that PM may play a more important role in determining mortality risk than the other pollutants [16,17]. This is a highly significant nationwide study conducted in a developing country, with substantial data accumulated over a long-term monitoring process. Moreover, it includes rural areas, which are usually out of normal monitoring, making the results more comprehensive and representative.

Globally, researchers have extensively explored the health effects of short-term exposure to air pollutants. The American College of Cardiology noted that exposure to PM_2.5_ for several hours to several weeks can lead to cardiovascular disease-related deaths and nonfatal events [18]. In a study based on the “Air Pollution and Health: a European Approach 2” project in 29 European cities, considering confounding and modification effects, researchers confirmed the short-term effects of atmospheric particulate matter on total mortality [19]. A systematic review of 196 articles revealed that short-term exposure to PM_10_, PM_2.5_, NO_2_, and O_3_ is positively correlated with all-cause mortality, whereas PM_10_ and PM_2.5_ are positively correlated with cardiovascular, respiratory, and cerebrovascular mortality [11]. Similar findings have been confirmed in many studies in China. Significant associations between short-term PM_2.5–10_ exposure and daily nonaccidental and cardiopulmonary mortality were found in a national analysis of 272 Chinese cities (0.25% increase in CVD incidence and 0.26% increase in respiratory disease incidence) [20]. In a time-stratified case-crossover study of 1,475,459 cardiopulmonary deaths in China, researchers demonstrated an association between short-term exposure to PM_2.5_ and cardiopulmonary mortality in the elderly [21]. The results of the time series analysis of CVD-related mortality in Jiangsu Province from 2015 to 2021 showed that each 10 μg/m^3^ increase in PM_2.5_ concentration was associated with a 0.72% increase in daily CVD mortality. Similarly, PM_10_ exposure increased daily mortality by 0.42% [22]. However, similar studies in China have focused mostly on urban meteorological monitoring sites in selected areas. Additionally, we found that the effect value of the association between short-term exposure to air pollutants and the risk of death in China is smaller than that reported in other countries. It has been suggested that there is a general tendency for PM_2.5_ to plateau significantly at high concentration levels in Chinese cities. In cities with chronically high PM_2.5_ levels, the health effects of increasing pollutant concentrations are smaller, namely the saturation effect of pollutants [23]. This is also possible because susceptible populations probably die before air pollutant concentrations reach significant levels [24]. The interpretation of the independent effects of pollutants in this study should be made with caution, because high correlations among the pollutants were observed in this study.

In stratified analyses that take into account participants’ characteristics, it is clear that the effect of increasing pollutant concentrations on the risk of death from CVDs and respiratory diseases in the population increases with age. This effect is even more pronounced for atmospheric particulate matter (PM_2.5_ and PM_10_), which can be linked to declining physiology in older people, who are likely to have some pre-existing chronic conditions [1,25]. A national study revealed that outdoor air pollution may contribute to the development of obesity in middle-aged and elderly people, which may indirectly contribute to the development and progression of CVDs and respiratory diseases [26]. In this study, we found that women have a greater risk of death due to air pollution, which is the same as the findings of certain researchers [27,28,29]. This regularity can be explained by differences in respiratory airway reactivity and deposition patterns of particulate matter between the sexes [29]. Due to anatomical and sex hormonal differences, males and females may differ in their susceptibility to diseases caused by air pollutants. Animal experiments have shown that female mice have more airway responsiveness [30]. As a predictor of airway health, high airway reactivity is characterized by airway narrowing and enhanced resistance which is often used to assess inflammatory airway diseases. In addition, particle deposition characteristics in the lungs vary by gender, with women having a higher proportion of all lung deposition, thereby increasing the absorption and metabolism of pollutants in the body [31]. These assumptions may explain the gender differences in mortality risk due to air pollution.

In terms of seasonal differences, we found that the increased risk of death due to pollution from particulate matter (PM_2.5_ and PM_10_) was more pronounced in the warm season. There may be a synergistic effect between air pollution and high temperatures, which can be explained by a heat stress response [32,33]. Increased ambient temperatures activate the respiratory and cardiovascular systems of the body [34], and this activation promotes the absorption and metabolism of toxic substances. However, we reached the opposite conclusion regarding the effect of temperature on the increase in deaths due to respiratory and CVDs caused by NO_2_. The conclusions are the same as those reached in studies conducted in Guangzhou, China [35,36]. The KORA F4 project in Augsburg, Germany, may offer an explanation [37]. There is a more pronounced correlation between lower temperatures and higher subclinical inflammatory biomarkers, which in turn cause more adverse effects on health through the activation of inflammatory pathways.

The finding of interest in this study is that the increased risk of death occurs more in urban areas than in rural areas. This may be related to concentrated industrial production, motor vehicle exhaust, more complex pollutant types (e.g., volatile organic compounds), high population density, and urban canyon effects [38,39]. China’s rapid urbanization is causing more and more people to live in areas with more severe air pollution [40]. Residents living in cities may face health threats such as shrinking green spaces and reduced physical activity, which can attenuate the body’s resistance to disease [41]. Some researchers have found a stronger association between CVD mortality and PM_2.5_ components [42]. In cities, complex fuel combustion and motor vehicle emissions lead to a mixture of gaseous and solid pollutants (particulate matter, PM), even generating secondary pollutants that are even more hazardous to human health. In addition, the particular geographic shape of cities makes the dispersion of pollutants more difficult [43]. However, with the modernization of rural production and the fact that protective measures against environmental pollution tend to be concentrated in urban areas, it is also clear that air pollution in rural areas has a nonnegligible impact on the health of the population [44]. It seems insufficient that most of the current research focuses on urban areas in developing countries. The present study included nationwide mortality data from urban and rural populations, which may allow for a more comprehensive estimation of the effects of short-term exposure to air pollution on the risk of death from respiratory diseases and CVDs. The results of this study show that the increase in the risk of death due to air pollution is more in urban areas than in rural areas. However, the effect value in rural areas is still significant. Therefore, there is still a need for environmental protection measures in rural areas to minimize population health damage.

In recent years, the Chinese government has significantly improved air quality by carrying out a series of measures to prevent and control air pollution. The current NAAQS no longer plays a dominant role in air quality regulation, as most areas are able to meet its stipulated standards. Although the values we obtained for the associated effects are low with a large population base and the accelerating phenomenon of aging, even a small risk of individual death can pose a significant threat to public health. The results of this study provide strong evidence for the short-term impact of some air pollutants on the mortality rates of respiratory diseases and CVDs and intend to play an active role in updating air quality standards and strengthening regulatory measures, especially in developing countries.

This approach has several significant advantages. First, this study collected and analyzed representative provincial-level data across the country, comprehensively revealing the associations between short-term air pollution exposure and mortality rates and providing a scientific basis for public health policies at the regional and national levels. Second, through the prediction of daily air pollutant concentrations via the NAQPMS, this study is able to assess the dynamic changes in air pollution in both spatial and temporal dimensions, significantly expanding the analysis scope traditionally reliant on data from fixed monitoring stations. Third, the extensive distribution of pollutants and long-term time series data collected between 2008 and 2020 provide this study with an unprecedented analytical capability to assess the impact of air pollution on mortality risk. Finally, the time-stratified case-crossover design employed in this study effectively controls for confounding factors, enhances the inferential strength of this study’s results, and provides a solid methodological foundation for the quantitative assessment of the health impacts of air pollution. This study also has certain limitations that need to be considered and improved upon in future research. First, we estimated the air pollution exposure levels of participants on the basis of the town/township/subdistrict (the smallest administrative unit in China) where they reside rather than their exact residential address. This approach may affect the precision of exposure assessment. Second, the design of this study did not cover the impact of long-term air pollution exposure on population health. Third, owing to the lack of detailed individual-level exposure data, we are unable to accurately identify and assess whether specific vulnerable groups (such as people with underlying diseases and economically disadvantaged populations) face greater health risks due to air pollution. Moreover, pre-existing conditions (e.g., cancer) might be registered as secondary causes of death, which could be a confounding factor in this study. Furthermore, we assessed the mortality risks associated with five major pollutants (PM_10_, PM_2.5_, NO_2_, CO, and SO_2_) in the present analysis. Given that growing studies indicate excessive ozone levels in China, and exposure to photochemical pollutants appears to be a major issue, the mortality risks posed by ozone should be investigated across urban and rural areas as well as in vulnerable populations. In further explorations, we intend to match pollutant exposure to more detailed address information of the study subjects; large-scale cohorts could be established to assess the effects of long-term pollutant exposure on population mortality; effect modification analyses can be considered to exclude the influence of confounding factors such as underlying diseases to further explore differences in health risks across populations.

## 5. Conclusions

This is a national case-crossover study with a long time span, wide spatial coverage, and a representative population. The results suggest that short-term exposure to air pollutants increases the risk of mortality from respiratory diseases and CVDs in both urban and rural populations in China. Estimates of effects in rural areas, although smaller than in urban areas, are still significant and might pose a greater health threat, especially in areas with large populations. These data reinforce the evidence of an association between cardiorespiratory mortality and air pollutants established in nationwide studies, which is particularly important for China and other low- and middle-income areas. In these regions, including all populations in both urban and rural areas, air pollution often poses a more significant threat due to limited resources for pollution control and less advanced environmental protection measures. Consideration could be given to improving national air quality management on the basis of WHO AQG levels, which would have far-reaching implications for protecting the health of populations, reducing the burden of disease, and promoting socio-economic development.

## Figures and Tables

**Table 1:** General characteristics of the study population.

Variables	Respiratory Diseases	CVDs
**Total**	7,171,833	30,339,986
**Sex**		
Male	4,201,902	16,613,491
Female	2,969,931	13,726,495
**Age (years)**		
<65	892,402	5,963,697
65–74	1,331,472	6,447,635
75–84	2,837,174	10,734,098
≥85	2,110,785	7,194,556
**Residential area**		
Urban	2,796,900	11,780,344
Rural	4,374,933	18,559,642
**Cause of death**		
Pneumonia	1,125,243	
Chronic obstructive pulmonary disease (COPD)	5,052,902	
Chronic bronchitis (CB)	1,090,813	
Emphysema	436,005	
Asthma	149,063	
Other Respiratory diseases	844,625	
Rheumatic heart disease (RHD)		448,467
Hypertensive heart disease (HHB)		2,982,601
Ischemic heart disease (IHD)		10,761,227
Cerebrovascular disease		13,741,489
Inflammatory heart diseases		150,351
Other CVDs		2,255,851

**Table 2:** Daily distribution of air pollution exposure levels on case and control days for two disease classifications.

Exposure	Respiratory Diseases		CVDs	
	Mean (SD), μg/m^3^	Median (IQR) [Difference], μg/m^3^	Mean (SD), μg/m^3^	Median (IQR) [Difference], μg/m^3^
**Case days**				
PM_2.5_	49.67 (35.84)	40.45 (41.44)	50.97 (37.21)	41.32 (43.25)
PM_10_	65.43 (44.72)	54.41 (52.86)	68.72 (46.65)	57.62 (56.42)
NO_2_	30.47 (22.59)	25.06 (30.87)	31.71 (22.49)	26.95 (31.31)
SO_2_	21.43 (21.12)	21.43 (20.47)	22.52 (22.21)	22.52 (21.99)
CO	0.78 (0.46)	0.78 (0.53)	0.79 (0.47)	0.79 (0.54)
**Control days**				
PM_2.5_	49.35 (35.75)	40.10 (41.20)	50.69 (37.14)	41.01 (43.06)
PM_10_	65.04 (44.59)	53.97 (52.50)	68.37 (46.56)	57.21 (56.15)
NO_2_	30.37 (22.53)	24.96 (30.76)	31.62 (22.44)	26.86 (31.22)
SO_2_	21.34 (21.05)	21.34 (20.40)	22.43 (22.15)	22.43 (21.91)
CO	0.78 (0.46)	0.78 (0.53)	0.79 (0.47)	0.79 (0.54)

Note: PM_2.5_, PM_10_, NO_2_, SO_2_ are measured in μg/m^3^, CO is measured in mg/m^3^.

**Table 3:** Percentage change in the risk of respiratory diseases and CVDs associated with increased ambient pollutant concentrations when controlling for copollutants (0-day lag).

Respiratory Diseases				CVDs			
	All Participants	Subgroup			All Participants	Subgroup	
		Urban	Rural			Urban	Rural
PM_2.5_	0.12 (0.09, 0.14)	0.20 (0.16, 0.24)	0.09 (0.05, 0.13)	PM_2.5_	0.08 (0.07, 0.09)	0.11 (0.09, 0.13)	0.07 (0.05, 0.08)
+NO_2_	0.12 (0.09, 0.15)	0.18 (0.12, 0.23)	0.09 (0.05, 0.14)	+NO_2_	0.10 (0.09, 0.12)	0.12 (0.10, 0.14)	0.10 (0.08, 0.12)
+SO_2_	0.14 (0.11, 0.17)	0.22 (0.17, 0.27)	0.10 (0.06, 0.15)	+SO_2_	0.10 (0.09, 0.12)	0.14 (0.12, 0.16)	0.09 (0.08, 0.11)
+CO	0.10 (0.07, 0.13)	0.18 (0.13, 0.23)	0.07 (0.03, 0.11)	+CO	0.07 (0.06, 0.09)	0.11 (0.09, 0.14)	0.06 (0.04, 0.07)
PM_10_	0.10 (0.08, 0.12)	0.16 (0.13, 0.19)	0.09 (0.06, 0.12)	PM_10_	0.07 (0.06, 0.08)	0.10 (0.08, 0.11)	0.06 (0.05, 0.08)
+NO_2_	0.11 (0.08, 0.13)	0.15 (0.11, 0.19)	0.10 (0.07, 0.14)	+NO_2_	0.09 (0.08, 0.10)	0.11 (0.09, 0.13)	0.10 (0.08, 0.11)
+SO_2_	0.11 (0.09, 0.14)	0.17 (0.14, 0.21)	0.11 (0.07, 0.15)	+SO_2_	0.09 (0.08, 0.10)	0.12 (0.10, 0.13)	0.09 (0.07, 0.10)
+CO	0.09 (0.07, 0.11)	0.15 (0.11, 0.19)	0.08 (0.05, 0.11)	NO_2_	0.03 (0.05, 0.14)	0.08 (0.05, 0.12)	0.02 (−0.01, 0.04)
NO_2_	0.10 (0.05, 0.14)	0.23 (0.16, 0.30)	0.10 (0.02, 0.17)	+PM_2.5_	−0.08 (−0.11, −0.06)	−0.04 (−0.08, 0.00)	−0.11 (−0.15, −0.07)
+PM_2.5_	−0.02 (−0.08, 0.03)	0.06 (−0.03, 0.15)	−0.01 (−0.09, 0.08)	+PM_10_	−0.10 (−0.12, −0.07)	−0.06 (−0.10, −0.02)	−0.13 (−0.17, −0.10)
+PM_10_	−0.03 (−0.09, 0.03)	0.05 (−0.04, 0.13)	−0.05 (−0.13, 0.04)	+CO	0.00 (−0.02, 0.02)	0.06 (0.03, 0.10)	−0.03 (−0.06, 0.04)
+SO_2_	0.10 (0.04, 0.16)	0.25 (0.16, 0.34)	0.09 (0.00, 0.18)	SO_2_	0.02 (0.00, 0.03)	0.03 (0.00, 0.05)	0.02 (0.00, 0.05)
+CO	0.06 (0.01, 0.11)	0.18 (0.01, 0.26)	0.05 (−0.03, 0.12)	+PM_2.5_	−0.08 (−0.10, −0.06)	−0.08 (−0.11, −0.05)	−0.08 (−0.12, −0.05)
SO_2_	0.05 (0.01, 0.10)	0.12 (0.05, 0.18)	0.08 (0.00, 0.15)	+PM_10_	−0.08 (−0.10, −0.06)	−0.08 (−0.11, −0.05)	−0.09 (−0.12, −0.06)
+PM_2.5_	−0.07 (−0.12, −0.02)	−0.06 (−0.14, 0.02)	−0.05 (−0.14, 0.05)	+CO	−0.01 (−0.03, 0.01)	0.01 (−0.02, 0.03)	−0.02 (−0.05, 0.01)
+PM_10_	−0.06 (−0.11, 0.06)	−0.05 (−0.12, 0.03)	−0.08 (−0.17, 0.01)	CO	0.22 (0.16, 0.28)	0.25 (0.15, 0.34)	0.30 (0.22, 0.38)
+NO_2_	−0.01 (−0.06, 0.05)	−0.02 (−0.11, 0.06)	0.02 (−0.08, 0.12)	+PM_2.5_	0.03 (−0.04, 0.10)	−0.04 (−0.15, 0.08)	0.14 (0.04, 0.24)
+CO	0.02 (−0.03, 0.06)	0.06 (−0.01, 0.13)	0.02 (−0.06, 0.10)	+NO_2_	0.22 (0.15, 0.28)	0.17 (0.06, 0.28)	0.34 (0.24, 0.43)
CO	0.40 (0.25, 0.54)	0.69 (0.45, 0.93)	0.45 (0.24, 0.66)	+SO_2_	0.23 (0.17, 0.30)	0.24 (0.13, 0.34)	0.32 (0.23, 0.42)
+PM_2.5_	0.14 (−0.02, 0.31)	0.22 (−0.06, 0.50)	0.28 (0.04, 0.51)				
+PM_10_	0.11 (−0.05, 0.28)	0.19 (−0.08, 0.47)	0.20 (−0.03, 0.44)				
+NO_2_	0.32 (0.17, 0.48)	0.45 (0.18, 0.72)	0.40 (0.17, 0.63)				
+SO_2_	0.38 (0.22, 0.53)	0.61 (0.35, 0.87)	0.43 (0.20, 0.66)				

Effect values are expressed as percent change and 95% confidence intervals. The two-pollutant model was fitted only when the correlation coefficient of the two pollutants was <0.70 [15].

## Data Availability

The original contributions presented in this study are included in this article/Supplementary Materials. Further inquiries can be directed to the corresponding authors.

## Supplementary Materials

The following supporting information can be downloaded at https://www.mdpi.com/article/10.3390/toxics13030156/s1: Table S1: Definitions of respiratory diseases and CVDs in the International Classification of Diseases 10th revision (ICD-10); Table S2: Percentage increases in the risk of death from respiratory diseases and CVDs on lag 0 due to short-term exposure to air pollutants; Table S3: Percentage changes in mortality risks from respiratory diseases and CVDs associated with increases in ambient pollutant concentrations stratified by the characteristics of the study participants. Table S4: Correlation coefficients for individual pollutants in respiratory diseases. Table S5: Correlation coefficients for individual pollutants in CVDs.
