# Supplementary material for: Short-Term Relationship Between Air Pollution and Mortality from Respiratory and Cardiovascular Diseases in China, 2008–2020
Source: Toxics. 2025 Feb 24;13(3):156. doi: 10.3390/toxics13030156 (PMC11946225; doi:10.3390/toxics13030156)
Supplement: Supplementary file 1 [file toxics-13-00156-s001.zip › toxics-3421458-supplementary.pdf]

Supplementary Table S1. Definitions of respiratory diseases and CVDs in the International Classification of Diseases 10th revision (ICD-10).

Disease	ICD-10
Respiratory diseases	J00-J99
Pneumonia	J12-J18
Chronic obstructive pulmonary disease (COPD)	J40-J44
Chronic bronchitis (CB)	J42
Emphysema	J43
Asthma	J45-J46
Cardiovascular diseases (CVDs)	I00-I99
Rheumatic heart disease (RHD)	I01-I09
Hypertensive heart disease (HHB)	I10-I13
Ischemic heart disease (IHD)	I20-I25
Cerebrovascular disease	I60-I69
Inflammatory heart diseases	I30-I33, I38, I40, I42

Supplementary Table S2. Percentage increases in the risk of death from respiratory diseases and CVDs on lag 0 due to short-term exposure to air pollutants.

	$PM_{2.5}$	PM_{10}	NO_2	SO_2	CO
**Total respiratory diseases**	0.12(0.09,0.14)	0.10(0.08,0.12)	0.10(0.05,0.14)	0.05(0.01,0.10)	0.40(0.25,0.54)
Pneumonia	0.27(0.20,0.35)	0.23(0.17,0.29)	0.26(0.13,0.40)	0.14(0.01,0.26)	1.06(0.63,1.48)
Chronic obstructive pulmonary disease (COPD)	0.11(0.08,0.14)	0.10(0.07,0.13)	0.16(0.10,0.21)	0.10(0.04,0.16)	0.47(0.29,0.66)
Chronic bronchitis (CB)	0.07(0.00,0.15)	0.08(0.02,0.15)	0.16(0.01,0.31)	0.09(-0.06,0.25)	1.16(0.68,1.65)
Emphysema	0.12(-0.01,0.24)	0.11(0.01,0.22)	0.38(0.13,0.64)	0.38(0.12,0.63)	0.98(0.16,1.81)
Asthma	0.10(-0.11,0.32)	0.10(-0.07,0.28)	0.33(-0.09,0.75)	0.29(-0.11,0.68)	0.75(-0.44,1.94)
**Total CVDs**	0.08(0.07,0.09)	0.07(0.06,0.08)	0.03(0.01,0.05)	0.02(0.00,0.03)	0.22(0.16,0.28)
Rheumatic heart disease (RHD)	0.05(-0.08,0.17)	0.10(-0.01,0.20)	0.19(-0.07,0.45)	0.13(-0.13,0.40)	-0.11(-0.91,0.69)
Hypertensive heart disease (HHB)	0.06(0.02,0.10)	0.08(0.05,0.12)	0.11(0.03,0.19)	0.07(-0.01,0.15)	0.36(0.12,0.59)
Ischemic heart disease (IHD)	0.10(0.08,0.11)	0.09(0.08,0.11)	0.05(0.02,0.09)	0.04(0.01,0.07)	0.34(0.23,0.45)
Cerebrovascular disease	0.10(0.08,0.11)	0.08(0.07,0.10)	0.08(0.05,0.11)	0.03(0.00,0.06)	0.33(0.24,0.43)
Inflammatory heart diseases	0.25(0.01,0.48)	0.27(0.08,0.45)	0.06(-0.38,0.50)	0.01(-0.43,0.45)	-0.25(-1.86,1.39)

Supplementary Table S3. Percentage changes in mortality risks from respiratory diseases and CVDs associated with increases in ambient pollutant concentrations stratified by the characteristics of the study participants.

	Respiratory diseases				
	$PM_{2.5}$	PM_{10}	NO_2	SO_2	CO
Age, years					
<65	0.03(-0.05,0.12)	0.06(-0.01,0.13)	0.08(-0.09,0.26)	0.08(-0.09,0.25)	0.58(0.00,1.15)
65-74	0.14(0.06,0.21)	0.15(0.09,0.20)	0.19(0.05,0.33)	0.17(0.04,0.31)	0.72(0.29,1.15)
75-84	0.15(0.10,0.19)	0.13(0.09,0.16)	0.19(0.11,0.27)	0.12(0.04,0.20)	0.74(0.49,1.00)
≥85	0.22(0.17,0.27)	0.19(0.15,0.24)	0.27(0.18,0.37)	0.15(0.05,0.24)	0.67(0.36,0.98)
Sex					
Male	0.10(0.07,0.14)	0.09(0.06,0.12)	0.13(0.07,0.19)	0.08(0.02,0.14)	0.52(0.31,0.72)
Female	0.18(0.13,0.22)	0.16(0.13,0.20)	0.19(0.11,0.27)	0.12(0.04,0.20)	0.59(0.33,0.86)
Season					
Cold	0.09(0.06,0.12)	0.07(0.05,0.10)	0.23(0.17,0.30)	0.11(0.06,0.17)	0.74(0.51,0.98)
Warm	0.30(0.24,0.35)	0.24(0.20,0.28)	0.09(0.01,0.17)	0.10(0.00,0.20)	0.46(0.24,0.69)
Residential area					
Rural	0.09(0.05,0.13)	0.09(0.06,0.12)	0.10(0.02,0.17)	0.08(0.00,0.15)	0.45(0.24,0.66)
Urban	0.20(0.16,0.24)	0.16(0.13,0.19)	0.23(0.16,0.30)	0.12(0.05,0.18)	0.69(0.45,0.93)
	**CVDs**				
	$PM_{2.5}$	PM_{10}	NO_2	SO_2	CO
Age, years					
<65	0.00(-0.03,0.02)	0.02(0.00,0.04)	0.00(-0.06,0.05)	-0.01(-0.05,0.04)	0.13(-0.04,0.30)
65-74	0.09(0.06,0.11)	0.09(0.07,0.11)	0.07(0.02,0.12)	0.04(0.00,0.09)	0.38(0.23,0.53)
75-84	0.11(0.09,0.13)	0.09(0.08,0.11)	0.08(0.04,0.11)	0.05(0.02,0.08)	0.37(0.26,0.47)
≥85	0.16(0.14,0.19)	0.15(0.13,0.17)	0.14(0.10,0.19)	0.07(0.03,0.11)	0.51(0.37,0.66)

Sex					
Male	0.06(0.04,0.07)	0.06(0.04,0.07)	0.02(-0.01,0.05)	0.01(-0.02,0.03)	0.18(0.10,0.27)
Female	0.12(0.10,0.13)	0.10(0.09,0.12)	0.08(0.05,0.11)	0.04(0.02,0.07)	0.38(0.29,0.48)
Season					
Cold	0.04(0.03,0.05)	0.03(0.02,0.04)	0.09(0.06,0.12)	0.04(0.02,0.06)	0.35(0.25,0.44)
Warm	0.23(0.20,0.25)	0.18(0.16,0.19)	0.01(-0.02,0.04)	0.04(0.01,0.08)	0.24(0.15,0.33)
Residential area					
Rural	0.07(0.05,0.08)	0.06(0.05,0.08)	0.02(-0.01,0.04)	0.02(0.00,0.05)	0.30(0.22,0.38)
Urban	0.11(0.09,0.13)	0.10(0.08,0.11)	0.08(0.05,0.12)	0.03(0.01,0.05)	0.25(0.15,0.34)

Supplementary Table S4. Correlation coefficients for individual pollutants in respiratory diseases.

	$PM_{2.5}$	PM_{10}	CO	NO_2	SO_2
$PM_{2.5}$		0.914	0.659	0.627	0.673
PM_{10}	0.914		0.686	0.636	0.622
CO	0.659	0.686		0.613	0.555
NO_2	0.627	0.636	0.613		0.699
SO_2	0.673	0.622	0.555	0.699	

Supplementary Table S5. Correlation coefficients for individual pollutants in CVDs.

	$PM_{2.5}$	PM_{10}	CO	NO_2	SO_2
$PM_{2.5}$		0.908	0.675	0.659	0.684
PM_{10}	0.908		0.701	0.657	0.634
CO	0.675	0.701		0.61	0.569
NO_2	0.659	0.657	0.614		0.732
SO_2	0.684	0.634	0.569	0.732	
